# Spatial utilization of historical topographic map and its application in land reconstruction of ancient Chinese urban land use

**DOI:** 10.1038/s41598-024-62493-2

**Published:** 2024-05-21

**Authors:** Zhiwei Wan, Hongqi Wu

**Affiliations:** 1 Department of History, Jinan University, Guangzhou, 510632 China; 2 School of Geography and Environmental Engineering, Gannan Normal University, Ganzhou, 341000 China

**Keywords:** Historical topographic map, Urban land, Spatial pattern analysis, Historical land use, Sustainability, Environmental sciences, Environmental social sciences, Planetary science

## Abstract

The historical topographic map preserves rich geographic information and can provide direct assistance for the reconstruction of various geographic elements. Based on the historical data of cities throughout the Qing Dynasty, the land use scale data of cities across the country was obtained using GIS and urban perimeter conversion models. This study combines city information and city circumference records from the historical maps and archives of the late Qing Dynasty to quantitatively reconstruct the use patterns of ancient China’s urban land at a spatial resolution of 1° × 1°. Uncertainty analysis of the reconstruction results was conducted using modern remote sensing image data as the validation data set. The results showed the following. (1) During the late Qing Dynasty, the total area of urban land in the various provinces and regions was 1456.015 km^2^. The maximum value was 208.691 km^2^ in Beijing-Tianjin-Hebei, the minimum value was 1.713 km^2^ in Qinghai, and the average value was 56.001 km^2^. (2) The results of grid reconstruction show that among the 398 grids with urban land distribution, the maximum value is 64.099 km^2^/grid, the minimum value is 0.013 km^2^/grid, and the average value is 3.658 km^2^/grid. (3) Of all the grids with urban land, the urban land grid to the west of the Hu Line accounts for 12.5% and the east to 87.5%. (4) During the late Qing Dynasty, urban land use in China was primarily concentrated in agriculturally developed areas such as the North China Plain, the Central Plains, Jiangnan, and the Sichuan-Chongqing region. (6) The results of a kernel density estimation showed that there were obviously three core areas of urban land agglomeration in China during the late Qing Dynasty: the North China Plain-Central Plains, the Jiangsu-Shanghai-Zhejiang-Anhui area, and the Sichuan-Chongqing urban core area. This study provides basic data for urban land use during historical periods and provides a basis for the quantitative reconstruction of relevant urban land data for historical archives.

## Introduction

The development pattern of cities in historical periods is of great help in helping us understand the process of urban development in modern society^1^. The pattern of modern urban development has a certain historical inheritance, and behind its development mechanism lies a certain development mechanism^2,3^. Understanding the spatial distribution pattern and characteristics of historical cities over a long period of time is of great significance for us to delve deeper into the process of modern urbanization^4,5^. Considering that China has a long urban history and development process, as well as rich historical literature, this also provides us with the possibility to explore the urban development process of over a century^6,7^. Due to China's thousands of years of unified history, it has developed a relatively mature urban and national governance system. This historical sample also provides a good reference material for urban and economic research. Research on the location selection and environmental background of cities during the ancient China shows that there is a coupling relationship between the size of cities and their administrative levels during the Chinese Empire period. In the urban system, as the city level increases, the importance of geographical features relative to institutional factors gradually weakens^8^. Therefore, obtaining a set of accurate data on the perimeter and area of cities at the national scale in the late Qing Dynasty is of great value for relevant historical and economic research.

In recent years, relevant scholars have established multiple sets of historical land use data sets at different scales. These include the *Sustainability and the Global Environment* (SAGE) of the University of Wisconsin^9^, the *History Database of the Global Environment* (HYDE) of Netherlands Environmental Assessment Agency^10^, and the *Chinese Historical Cropland Dataset* (CHCD) of Chinese Academy of Sciences^11^. The development of these data sets provides comparative data for model boundaries and accuracy verification for the quantitative measurement of ecological pattern changes^12^. Currently, the reconstruction of various types of land uses during the historical period of China has concentrated on cultivated land^13–15^, forests^16^, and grasslands^17,18^. Some studies have also begun to conduct comprehensive land use reconstructions for historical periods^19,20^ that include rocky desertification^21^ and the reconstruction of construction land at the provincial scale^22^. However, the system reconstruction of urban land use at the national scale during the past 100 years has been limited due to a lack of data, and this effort is currently in development. Urbanized areas are areas where humans have transformed the surface morphology on a large scale. Therefore, it is of great significance to quantitatively reconstruct the urbanization pattern of the historical period. Remote sensing image data can generally only be used for urban land use reconstruction of the past 40 years^23,24^. In addition, to restore the urban land scale and architecture of the past 100 years requires new data sources.

In previous studies, the reconstruction of land use has almost always been based on the assumption that the ancient urban land use pattern may be consistent with the modern urban pattern^25^, and this has been used to reconstruct historical urban land use patterns retrospectively^26,27^. The background and basis of the development of ancient Chinese cities are different from that of modern China, especially the pattern of Chinese cities that have developed rapidly after the reform and opening up of the nation. Therefore, there is an urgent need for a more reasonable method to reconstruct the area and patterns of urban land use in China during the historical period^28–30^.

In the research of urban land use in historical periods, limited by data sources and accessibility, most of the current research uses remote sensing data for nearly 40 years of urban land use research in China. In order to extend the research time series, some scholars have also started using keyhole satellite data to study the urban land use situation in China since the 1960s. Due to data limitations in China, urban land use research before the 1950s often could only be analyzed based on a small amount of aerial photography data and cadastral survey records from cities. Considering the above limitations, there is an urgent need to introduce new historical data, especially data with precise surveying, for analyzing urban land use patterns at the national scale.

China has relatively complete cultural classic and archive records. In particular, the Qing Dynasty rulers compiled a series of large-scale local chronicle books^31^. Among them, the *Emperor Jiaqing Reconstruction of Unification Chronicles*, which was written in the middle and late Qing Dynasty (completed in AD 1842), records the perimeter of the city wall, the basic situation of the city, the administrative level, and other information about cities above the county level in China^32^. Based on this set of data, He et al.^33^ conducted an estimation and preliminary study on the urban land areas of 18 provincial administrative regions in the hinterland during the Qing Dynasty. In recent years, with the utilization of surveying and mapping materials from the late Qing Dynasty and the study of other archival materials, the perimeter records of the city walls of the Qing Dynasty began to be studied by relevant scholars^2,34^. This study is based on archival data and mapping data from the late Qing Dynasty. Considering that this set of historical materials was published in 1842, the city information collected should represent the situation before 1842. Therefore, this study sets the research period as the 1840s. This study then uses the geographic information system and grid analysis methods, combined with methods such as the kernel density estimation and Moran’s index, to build the ancient china urban land pattern during the late Qing Dynasty with a resolution of 1° × 1°. Because these data sets are supported by real archival data combined with the cross-validation of modern remote sensing image data that retains the ancient city walls today, the uncertainty of the data set is reduced. Therefore, this study provides land use verification data for future urbanization research and various large-scale ecological models of the historical period.

## Materials and methods

### Study area

**Figure 1:**
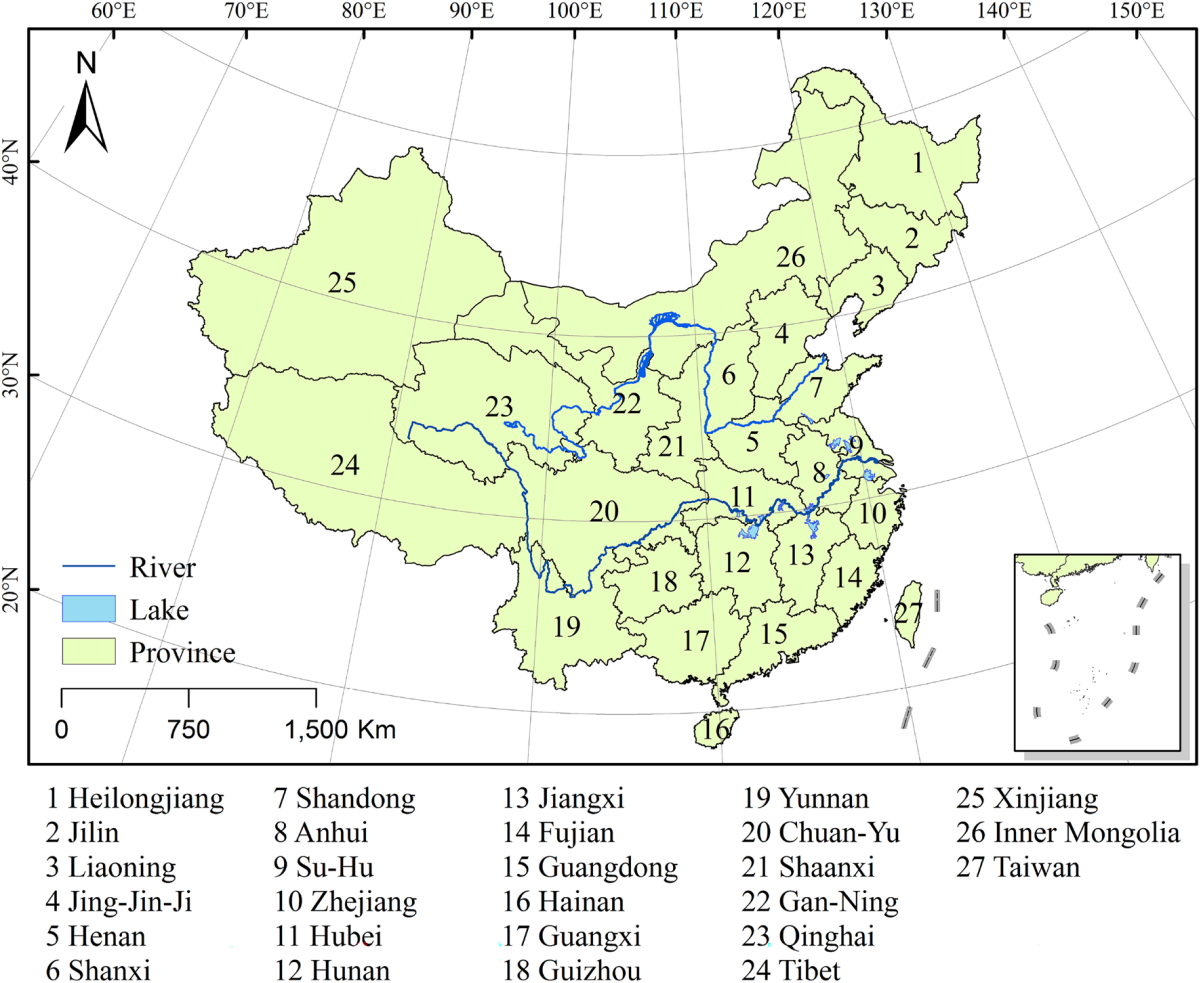
Study of the regional administrative divisions and schematic maps (The map was drawn using ArcGIS 10.2 ESRI, Redlands CA).

The study area was chosen based on the modern China administrative divisions (Fig. 1) combined with the administrative divisions of the late Qing Dynasty. The study area was divided into 27 provincial administrative areas that included Heilongjiang, Jilin, Liaoning, Inner Mongolia, Qinghai, Tibet, Xinjiang, and other provinces that belonged to the traditional frontier region^35^. Based on the actual physical geographic situation of the provinces and regions, Beijing, Tianjin, and Hebei were merged into the Jing-Jin-Ji region. Jiangsu and Shanghai were merged into the Su-Hu region. Sichuan and Chongqing were merged into the Chuan-Yu region, and Gansu and Ningxia were merged into the Gan-Ning region^36^. The reason why regional consolidation was carried out according to this principle is mainly due to the fact that these regions have historically belonged to the same administrative region, and have relatively consistent natural geographical stripes and spatial continuity. For example, Beijing Tianjin Hebei was under the jurisdiction of the Governor General of Zhili during the Qing Dynasty, Shanghai was part of Jiangsu Province during the Qing Dynasty, Gansu and Ningxia were also under the same provincial-level administrative region during the Qing Dynasty, and Chongqing was part of Sichuan during the Qing Dynasty. In addition, due to the special natural geographical conditions of the plateau in Tibet, its urban development was restricted in history, and there exist relatively few records regarding its cities in related historical archives^37^. Moreover, the organizational form of the cities in the plateau area was also quite different from that of ordinary cities, so this study regarded it as no urban area. In Taiwan, due to a lack of relevant files, this study temporarily treated it as a data-free area.

### Research materials

#### Historical records on the city wall

**Figure 2:**
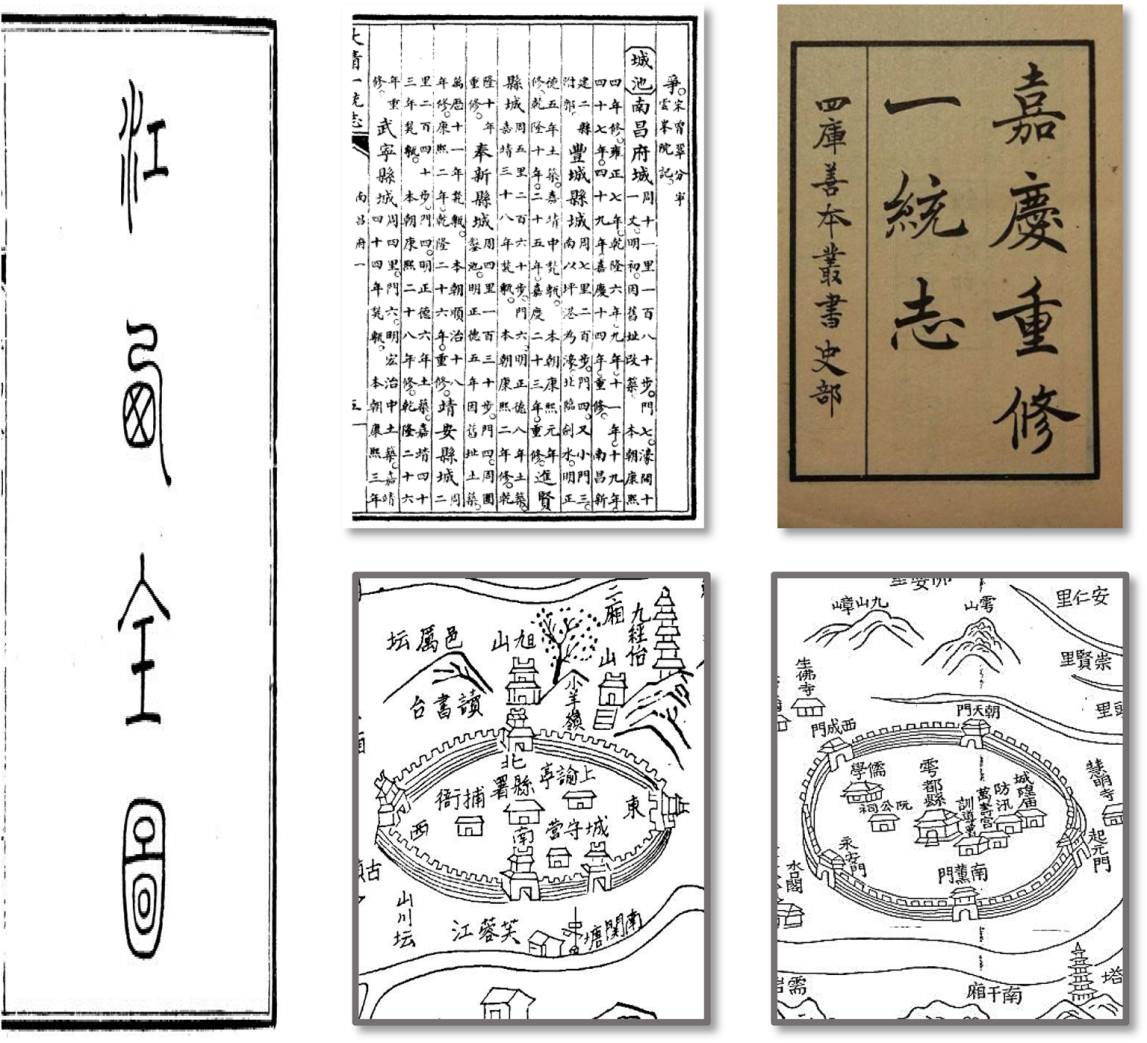
Legend of the city and wall from the Emperor Jiaqing reconstruction of unification chronicles.

The data on the perimeter of the city used in this research were obtained from the *Emperor Jiaqing Reconstruction of Unification Chronicles* completed in AD 1842^32^. These files primarily include cities above the county level in the provinces, except for the border regions. This set of files has a detailed description of the information about the city administrative level, the nature of the city, and the perimeter of the city wall. Some cities are equipped with maps, but such maps are often descriptive pictures without actual survey and mapping information (Fig. 2). The data on the perimeter of cities in the frontier areas primarily originate from the statistical literature of other scholars^38–40^.

#### Historical military topographic map

For cities with missing records or cities with measured topographic maps during the late Qing Dynasty, the geographic information system was used directly for area measurements and reconstruction. The military topographic maps from the late Qing Dynasty were primarily derived from the maps of the provinces of the entire country collected by the Institute of Modern History, Academia Sinica in Taiwan (download address is http://map.rchss.sinica.edu.tw/). The main time distribution of this set of topographic maps is between 1860 and 1890s.

**Figure 3:**
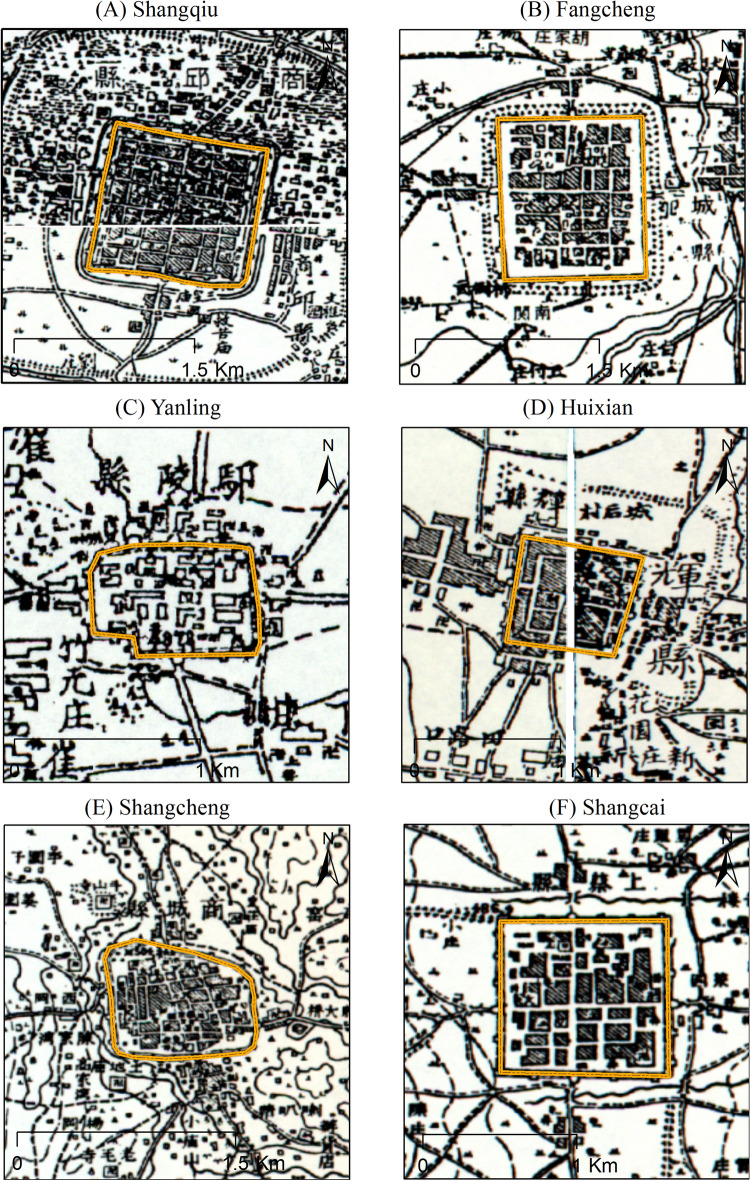
Military topographic map data of the city in the late Qing Dynasty (**A**) Shangqiu; (**B**) Fangcheng; (**C**) Yanling; (**D**) Huixian; (**E**) Shangcheng; (**F**) Shangcai (The yellow line indicates the city wall).

The cities in the late Qing Dynasty used vector files in the historical map data sets of Harvard University and Fudan University for positioning, while referring to the modern city location and remote sensing data for spatial processing. The urban spatial information in the historical period comes from the CHGIS-1911 data set (http://www.people.fas.harvard.edu/~chgis/data/chgis/v6/). All data processing and calculation are done in ArcGIS 10.2 platform. For some cities with measured topographic maps during the late Qing Dynasty (Fig. 3), the surveying and mapping information in the topographic maps can be used for vectorization and spatial georeferencing in ArcGIS 10.2. By following the above steps, the scope of the city wall can be drawn and the urban area can be calculated.

### Research methods

#### Method of calculating the area: the ancient city treated as a square

This study used the quadrature method proposed by He et al.^33^ to convert the circumference and area of ancient Chinese cities. In this method, the city is regarded as a square, so the perimeter of the city recorded in the historical archives was used to calculate the area of the city. The conversion formula is

1 $$ Ai{ = }\left( {Ci/4} \right)^{2} , $$

where *A*_*i*_ is the area of the *i*-th city; and *C*_*i*_ is the circumference of the *i*-th city.

For the length unit in the archive records of the Qing Dynasty: *li* is a unified conversion of 1 *li* = 576 m^41^. Although there is a certain deviation in the unified treatment of the shape of ancient cities directly according to squares, relevant studies have shown that^22,33^, due to the construction technology, most cities in ancient China are squares or approximate squares. This conversion principle has been applied in some practical applications, and the resulting deviations have not been large.

#### Spatial grid statistics

Using the fishing net generation function in ArcGIS 10.2 software, the research area has meshed with a resolution of 1° × 1°. The statistics of urban land area are calculated according to the province area and grid respectively, and the relevant calculation process is completed in ArcGIS 10.2.

#### Spatial pattern analysis

The spatial pattern of urban land use can be identified by the global Moran’s I. It is generally believed that the value of I is in the range of [− 1, + 1]. The closer to − 1, the higher the negative correlation of the spatial distribution of things; the closer to + 1, the higher the positive correlation^42,43^. The relevant formula is:

2 $$ {\text{Moran}}^{\prime}s{\text{ I = }}\frac{n}{{\sum\nolimits_{ij}^{n} {wij} }} \times \frac{{\sum\nolimits_{ij}^{n} {w_{ij} (xi - \overline{x} )(xj - \overline{x} )} }}{{\sum\nolimits_{i}^{n} {(xi - \overline{x} )^{2} } }}, $$

where, *x*_*i*_ and *x*_*j*_ are sample values which represents the urban area of each grid, $\overline{x}$ is the mean, *w*_*ij*_ is the spatial weight matrix.

Local Moran’s I statistics^44^ can better reflect the aggregation status and patterns of individual independent spatial units, so they are often used to identify cold and hot spots in space. This method finds high-high adjacent areas, that is, hot spots in urban land use. The formula for the local Moran’s I is:

3 $$ {\text{Local Moran}}^{\prime}s{\text{ I = }}\frac{{n(xi - \overline{x} )\sum\nolimits_{j} {w_{ij} (xj - \overline{x} )} }}{{\sum\nolimits_{j}^{{}} {(xi - \overline{x} )} }}, $$

where *n* is the number of grids, *w*_*ij*_ is the spatial weight matrix, *x*_*i*_ and *x*_*j*_ are sample values, $\overline{x}$ is the mean.

Kernel density estimation is a non-parametric test method^45^, which can be used to analyze the density of spatial point distribution^46^. The basic principle is to estimate the theoretical distribution of the sample points in the area through the kernel density function, and convert the discrete sample point density into a spatially continuous density value. Kernel density analysis can identify the concentrated areas of spatial point element distribution, that is, hotspot distribution areas. The calculation method is:

4 $$ F_{n} (x) = \frac{1}{nr}\sum\nolimits_{i = 1}^{n} {k\left( {\frac{{x - x_{i} }}{r}} \right),} $$

where *k*(·) is the kernel function, *r* is the analysis radius, and $x - x_{i}$ is the distance between the point *x* to be estimated and the sample point *x*_*i*_.

#### Research workflow

This study first systematically collected the perimeter of cities above county level in the late Qing Dynasty historical materials (*Emperor Jiaqing Reconstruction of Unification Chronicles* completed in AD 1842), and converted the perimeter into area according to Eq. (1) to obtain a dataset of urban land area for cities above county level nationwide. Then, using ArcGIS software, the total urban area in each grid is obtained through grid partitioning. Finally, through relevant analysis and statistical methods, the spatial pattern analysis of urban land use in China in the late Qing Dynasty was completed.

## Results

### Urban land area at the provincial scale

**Figure 4:**
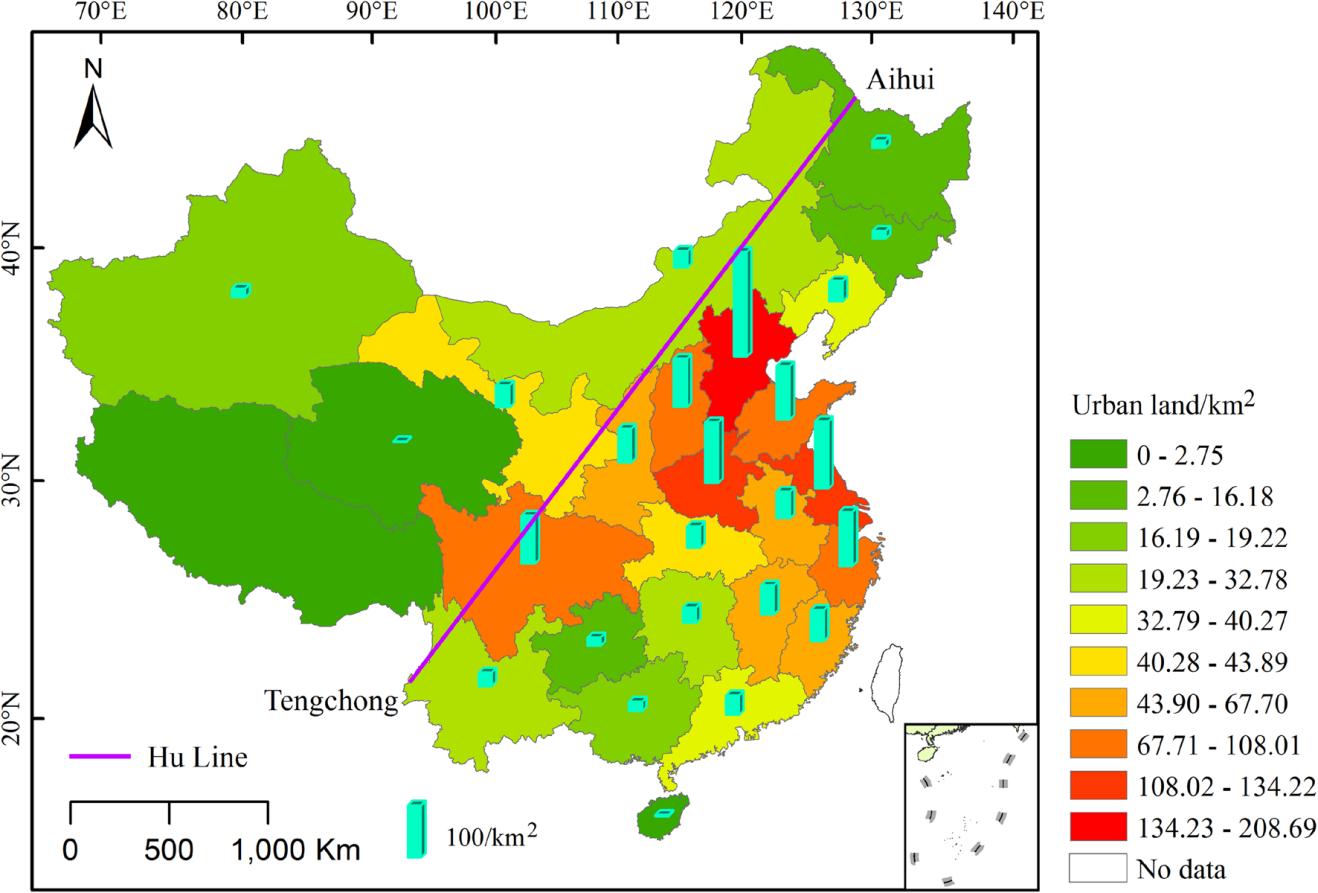
Distribution of land use areas in China cities during the late Qing Dynasty at the provincial scale (The map was drawn using ArcGIS 10.2 ESRI, Redlands CA).

The calculation results show that the total area of urban land in the various provinces and regions in China during the late Qing Dynasty was 1456.015 km^2^. The top five with the largest areas were Jing-Jin-Ji (208.691 km^2^), Su-Hu (134.218 km^2^), Henan (120.309 km^2^), Zhejiang (108.008 km^2^), and Shandong (106.436 km^2^). The top five with the smallest areas were Qinghai (1.713 km^2^), Hainan (2.754 km^2^), Heilongjiang (15.051 km^2^), Jilin (15.722 km^2^), and Guizhou (16.179 km^2^). It can be seen from Fig. 4 that during the late Qing Dynasty, there was a large gap in the area of urban land use among the provinces and regions in China. Except for Taiwan and Tibet, where the relevant data were lacking, the average area of urban land use in each province was 58.240 km^2^, the standard deviation was 49.331, and the coefficient of variation was 0.881. Moreover, it can be seen that the area of urban land use differs between the provinces and regions on both sides of the Hu line, and most of the urban land was distributed in the southeast direction of the Hu line.

### Urban land area at the grid scale

**Figure 5:**
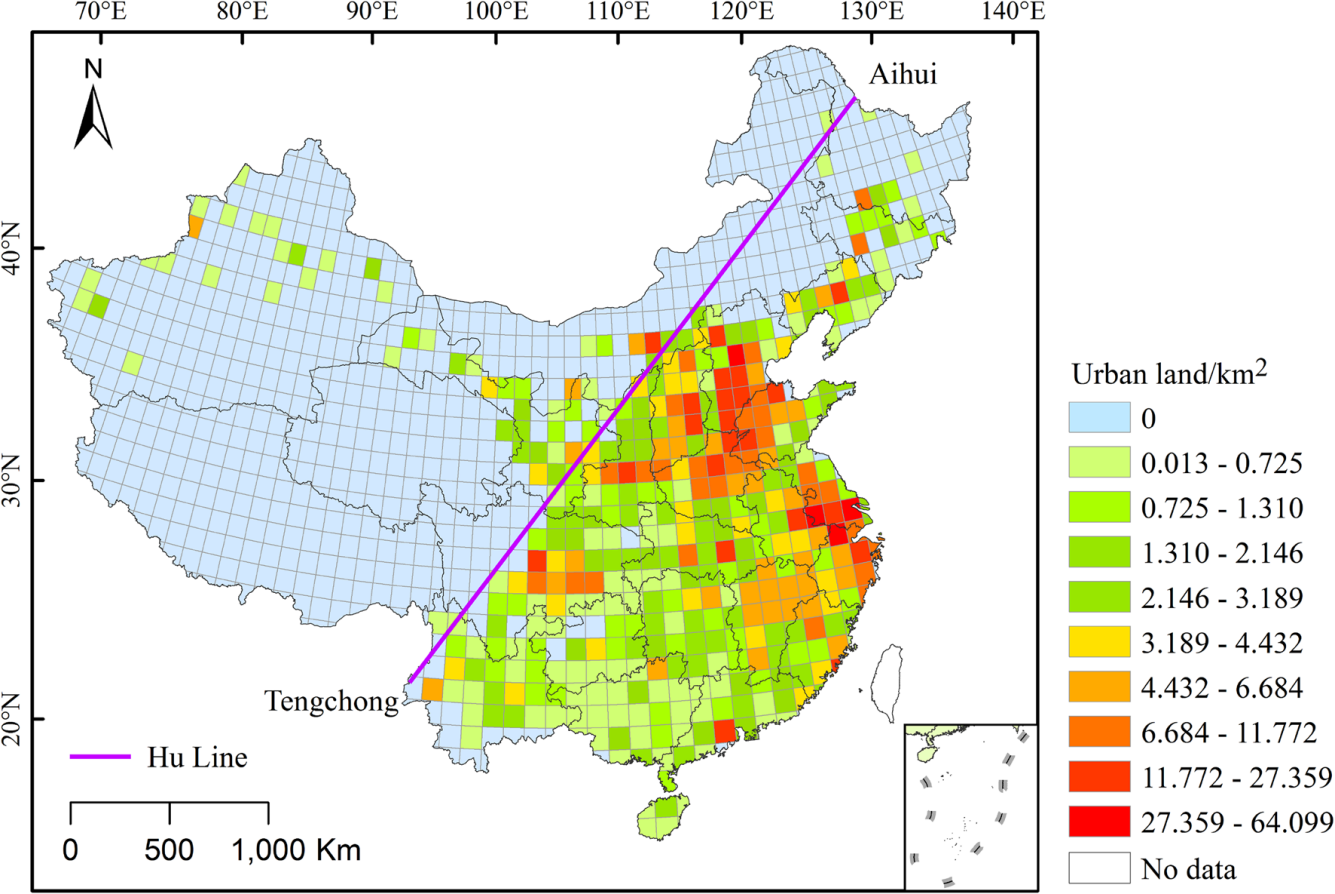
Distribution of the land use area of China’s cities during the late Qing Dynasty at the 1° × 1° grid scale (The map was drawn using ArcGIS 10.2 ESRI, Redlands CA).

The 1° × 1° resolution grid urban land use reconstruction results are displayed in Fig. 5. Of all the 1096 grids, 698 have an urban land area of 0. Among the remaining 398 grids, the maximum value is 64.099 km^2^, the minimum value is 0.013 km^2^, and the average value is 3.658 km^2^. It can be seen from Fig. 5 that during the late Qing Dynasty, urban land use in China was primarily concentrated in agriculturally developed areas, such as the North China Plain, Central Plains, Jiangnan region, and the Chuan-Yu region. Taking the Hu Line as the boundary, only 50 of the 398 grids with urban lands are located northwest of the Hu Line, accounting for only 12.5%. This distribution pattern is related to the plain topography and more developed agriculture in the North China Plain and the Central Plains of China, which can support larger cities. The larger cities in the Jiangnan region are more related to the region’s history of a commodity economy and agriculture, and their urbanization level has always been greater. In the context of the lower scale of cities in the west and south, the larger urban land use areas of regional cities, such as the Sichuan Plain and the Pearl River Delta, are obviously related to the plain of the terrain, developed agriculture, and commerce.

### The spatial distribution pattern

**Figure 6:**
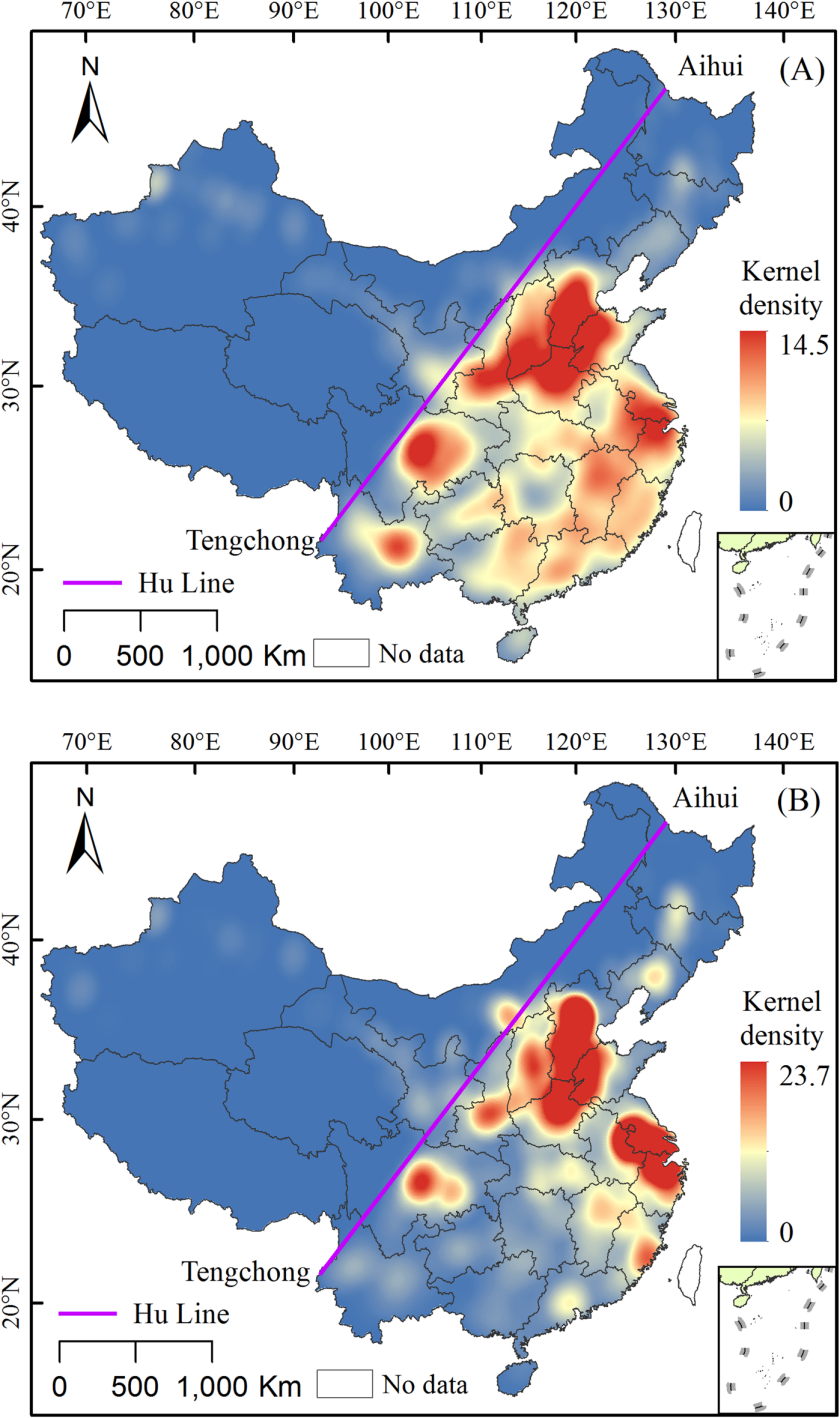
Kernel density map of the urban spatial distribution in China during the late Qing Dynasty (**A**) and the kernel density map of the urban land use area (**B**) (The map was drawn using ArcGIS 10.2 ESRI, Redlands CA).

The results of the kernel density estimation on urban spatial distribution indicated that the Chinese cities during the late Qing Dynasty were primarily distributed in the southeast of the Hu Line (Fig. 6A), of which the North China Plain, Central Plains, and Guanzhong Plain are the largest urban distribution areas with high kernel density values. Second, Su-Hu, Anhui, Zhejiang, Jiangxi, and other regions, as well as the Sichuan Basin, are the next-level high-density areas. In addition, some small areas of high kernel densities formed in the Pearl River Delta and Yunnan. When the kernel density analysis was conducted after considering the area of urban land use, it was found that there were obviously three core areas of urban land use in China during the late Qing Dynasty (Fig. 6B). They were the North China Plain and the Central Plains urban core area; the Su-Hu, Zhejiang, and Anhui regions; the Jiangnan urban core area; and the Chuan-Yu urban core area.

### Grid reconstruction of the urban land use

**Figure 7:**
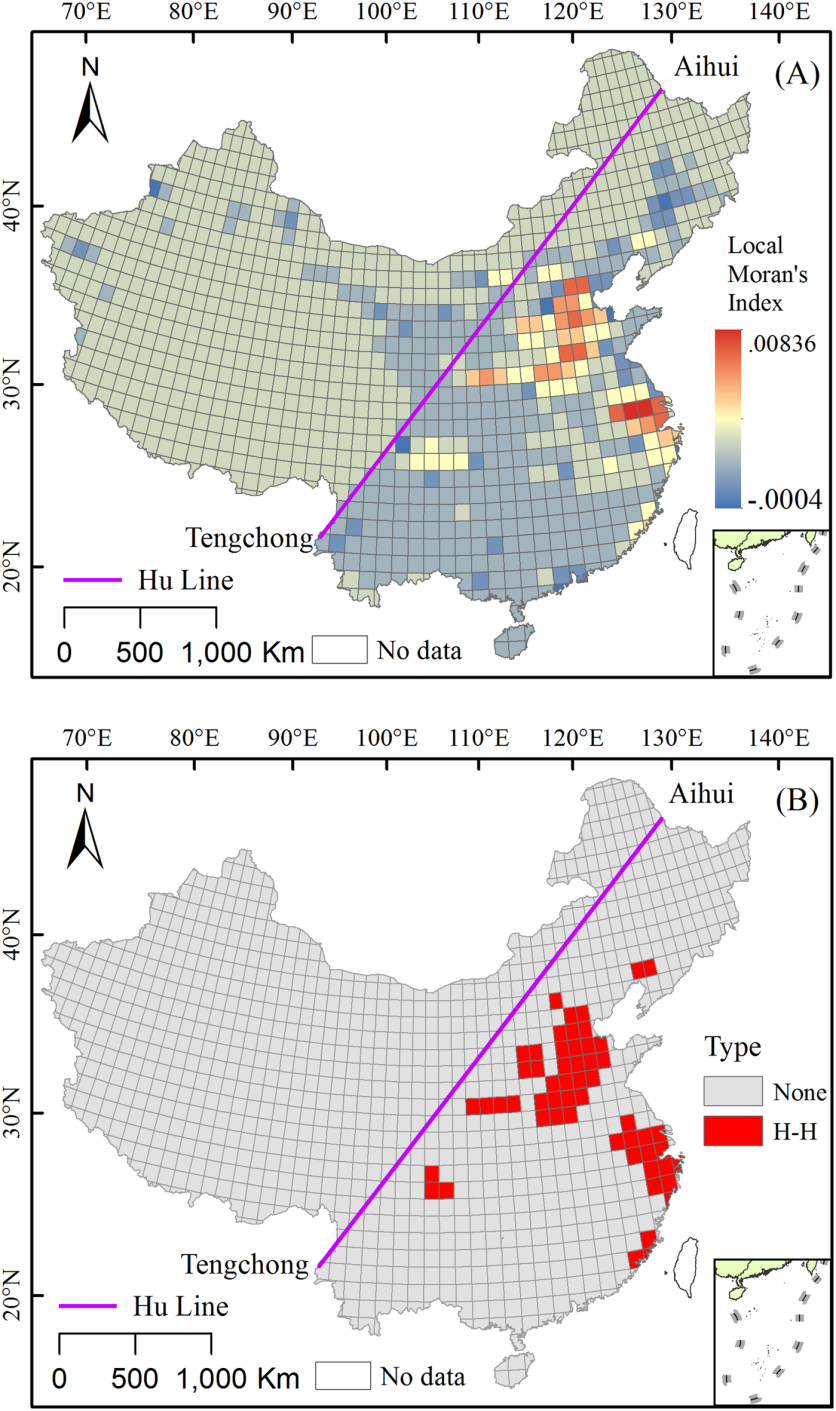
Local Moran’s I value distribution map (**A**) and spatial agglomeration type (**B**) of the scale of urban land use in China during the late Qing Dynasty (The map was drawn using ArcGIS 10.2 ESRI, Redlands CA).

With the support of ArcGIS 10.2 and GeoDa 0.9.5 software, the global Moran’s I index of the city scale in the late Qing Dynasty was calculated to be 0.346 (Z-Score = 13.502, P < 0.001). This indicates that its spatial distribution has obvious agglomeration characteristics, that is, the high value area of the urbanization level is adjacent to the high value area (H–H), and the low value area is adjacent to the low value area. The results of the local Moran’s I (Fig. 7A) showed that the high-value areas were primarily distributed in Jing-Jin-Ji, Henan, and Su-Hu. The results of the cluster type analysis showed (Fig. 7B) that the clusters of high-value areas represented by H–H were primarily distributed in the southeast of the Hu line. Except for a small number of H–H districts distributed in Liaoning, Chuan-Yu, and Fujian, most of the H–H districts were located in the provinces with large-scale urban lands, such as the Jing-Jin-Ji, Henan, Shaanxi, and Jiangnan regions.

From the perspective of historical development, cities in historical periods need to be built on the foundation of agricultural development. Most of the cities in traditional China belonged to administrative cities, which together with the rural areas they managed formed a system. Therefore, the spatial distribution pattern of cities in Chinese history was influenced by agricultural activities, especially the spatial distribution pattern of cultivated land. China has a long history, with agricultural activities originating from the Yellow River Basin in the central region and gradually developing towards the Yangtze River Basin and the south. These major agricultural development areas basically overlap with the core urban spatial distribution areas identified in our research. It can be seen that cities, as a part of the entire agricultural system, have a high degree of overlap between the two subsystems.

## Discussion

### Uncertainty analysis: comparison with remote sensing data

**Figure 8:**
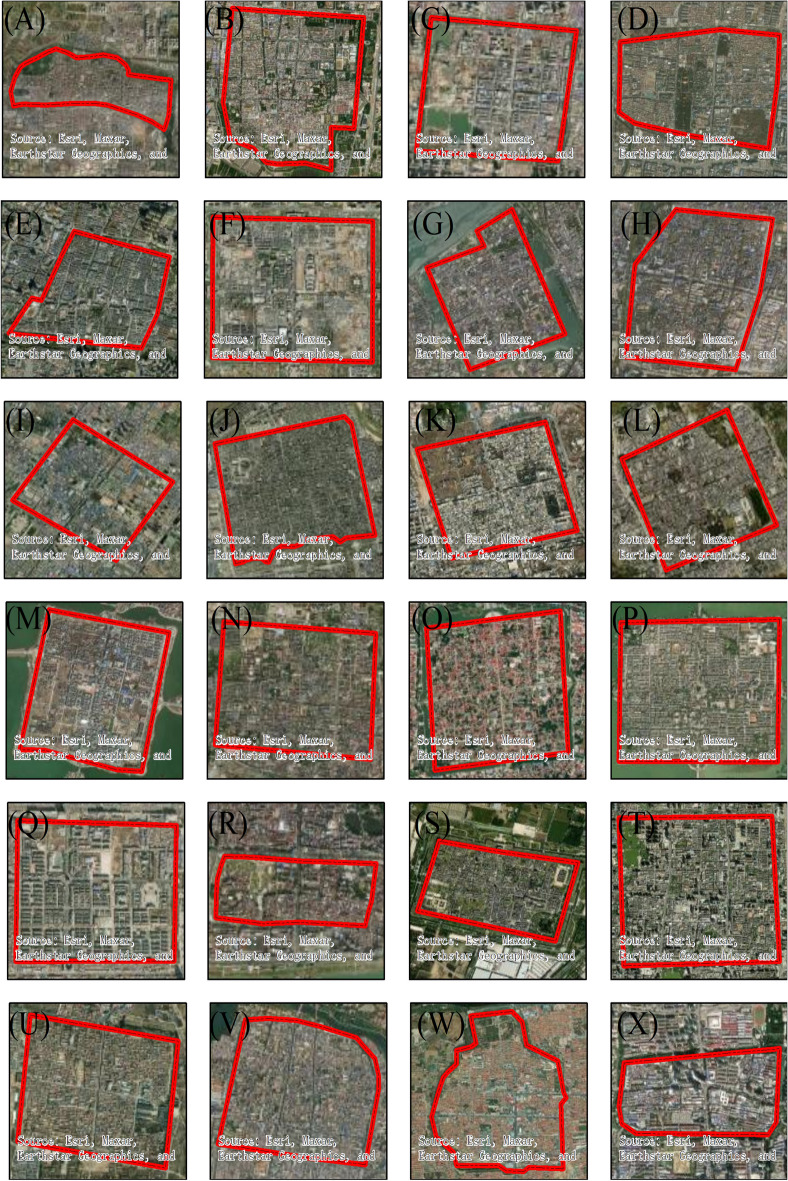
Urban remote sensing image that retains the city wall of the Qing Dynasty (The red line indicates the city wall).

**Figure 9:**
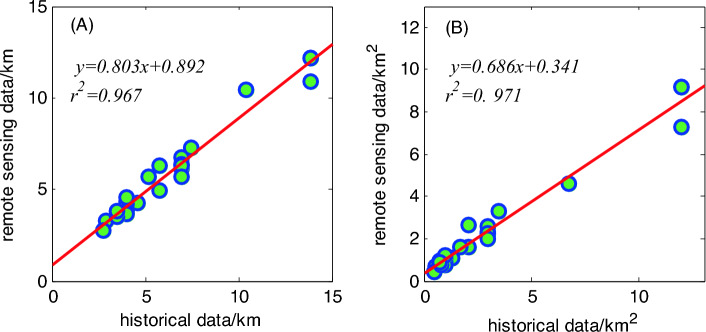
Correlation between historical data and remote sensing data. (**A**) City circumference data; and (**B**) city land use area data.

The reconstruction of the area of ancient urban land based on the perimeter of the city and the square quadrature formula will have certain errors. Therefore, to more effectively explain the reliability and error range of the reconstruction results, this study system sorted out the ancient cities in China that have relatively complete Qing Dynasty city walls and used Google Earth’s remote sensing image data to measure the circumference and area of the cities. Thereby, a set of verification data was obtained. The set of remote sensing survey datasets for Qing Dynasty’s cities included 24 cities (Fig. 8): Jingzhou, Zhengding, Heze, Qufu, Baoding, Datong, Xiangyang, Nanyang, Fenyang, Pingyao, Xingcheng, Dali, Shangqiu, Xuanhua, Daming, Liaocheng, Shuozhou, Zhaoqing, Taiyuan, Yangqu, Wanquan, Yuzhou, Yuxian, and Suzhou. The walls of these cities during the Qing Dynasty are well preserved and can be interpreted from remote sensing images. The perimeters and urban land areas of the cities based on the remote sensing image measurements can be considered as true values. Therefore, these results were obtained using a regression analysis of the historical data and remote sensing data. The correlation coefficient between the length of the ancient city wall reconstructed based on historical data and the remote sensing measurement data was 0.983 (Fig. 9A), and the regression equation was *y* = 0.803*x* + 0.892 (*r*^2^ = 0.967, p < 0.001). The correlation coefficient between the urban area reconstructed based on historical data and the remote sensing measurement data was 0.985 (Fig. 9B), and the regression equation was *y* = 0.686*x* + 0.341 (*r*^2^ = 0.971, p < 0.001). It can be seen that the reconstruction results are highly correlated with the remote sensing data, and the quadrature method proposed by He et al.^33^ more effectively reconstructed urban land use during the historical periods.

### Comparison with other studies

According to our previous research on the reconstruction of urban scale in historical China, the total area of county-level and above cities in China in the 1930s was 1396.48 km^2^^3^. The reason for this difference is that these two studies belong to different time periods. One article was conducted in the 1930s, while this study was conducted in the 1840s. On the other hand, these two studies utilize different data. The urban data of the 1930s came from actual military topographic maps and belonged to the actual urban land scale. And this study is based on the urban perimeter data recorded in historical materials, converted into urban land area according to the conversion equation, so it has certain differences from the actual mapping data.

**Figure 10:**
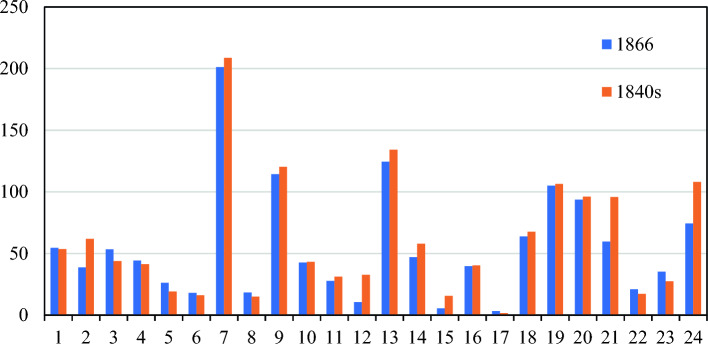
Comparison of Two Datasets (The urban area data for 1866 is sourced from Xue et al. ^47^, 1:Anhui, 2:Fujian, 3:Gan-Ning, 4:Guangdong and Hainan, 5:Guangxi, 6:Guizhou, 7:Jing-Jin-Ji, 8:Heilongjiang, 9:Henan, 10:Hubei, 11:Hunan, 12: Inner Mongolia, 13:Su-Hu, 14:Jiangxi, 15:Jilin, 16:Niaoling, 17:Qinghai, 18: Shaanxi, 19: Shandong, 20: Shanxi, 21:Chuan-Yu, 22:Xinjiang, 23:Yunnan, 24:Zhejiang).

**Figure 11:**
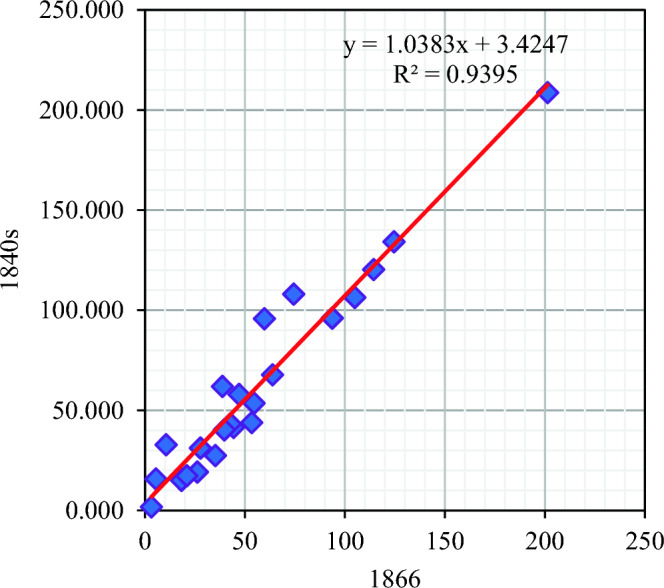
Results of regression analysis on two datasets.

Xue et al.^47^ provided urban reconstruction data for China in the nineteenth century. We compared the results of this study with them and found that there was some consistency between the two datasets. Due to missing data, this study did not collect relevant data for Taiwan Province. Therefore, after summarizing the data from Xu et al. the total area of cities in China in 1866 was 1323.130 km^2^, while the total area of cities in China in the 1840s obtained in this study was 1456.015 km^2^ (Fig. 10). The results of regression analysis show that there is a significant correlation between the two datasets (Fig. 11), with a correlation coefficient of 0.969 (p < 0.001).

### Limitations of the study

The long-term urban land use reconstruction work requires relevant data and materials as support, especially on a national scale, which makes it difficult to obtain such detailed data. The current sources of remote sensing image data, cadastral statistical data, and topographic maps are difficult to complete large-scale urban land use reconstruction work that has exceeded 100 years. This study attempts to use official historical materials from the late Qing Dynasty to extract perimeter data of cities at or above the county level nationwide, and uses a transformation model to obtain a dataset of land use scale of cities at or above the county level nationwide in the 1840s. Although this dataset has certain errors in specific urban area reconstruction, the overall reconstruction of the spatial distribution pattern of urban scale still has strong scientific significance. According to the analysis results of the validation data, the overall error is relatively small and within an acceptable range. This research achievement can provide a national scale long-term series reference for the study of urban land use in historical periods.

## Conclusions

The long-term scale of urban land use and its spatial pattern in Chinese history is of great help for us to understand the process of urban development in China. This study quantitatively reconstructed the spatial distribution pattern of urban land use in traditional China during the late Qing Dynasty using historical literature and model transformation methods. The analysis of the spatial pattern characteristics of national urban land use during this historical period is of great significance to related disciplines. At the same time, the research results also demonstrate the historical basis of the spatial distribution of modern cities in China.

1. During the late Qing Dynasty, the total scale of urban land in the various provinces and regions in China was 1456.015 km^2^. The top 5 with the largest area are: Jing-Jin-Ji (208.691 km^2^), Su-Hu (134.218 km^2^), Henan (120.309 km^2^), Zhejiang (108.008 km^2^) and Shandong (106.436 km^2^); the top 5 with the smallest area are: Qinghai (1.713 km^2^), Hainan (2.754 km^2^), Heilongjiang (15.051 km^2^), Jilin (15.722 km^2^) and Guizhou (16.179 km^2^).
2. The 1° × 1° resolution grid reconstruction results showed that, out of all 1096 grids, 698 had an urban land area of 0. There were 398 grids with urban land distributions, the maximum value was 64.099 km^2^/grid, the minimum value was 0.013 km^2^/grid, and the average value was 3.658 km^2^/grid.
3. By using the Hu Line as the dividing line, only 50 of the 398 grids with urban lands were located in northwest of the Hu Line, accounting for only 12.5%. During the late Qing Dynasty, urban land use in China was primarily concentrated in agriculturally developed areas, such as the North China Plain, Central Plains, Jiangnan, and Chuan-Yu.
4. The perimeter data of cities in the historical archives were converted to the area using the quadrature method. In a comparison with the remote sensing data, the correlation coefficient between the reconstructed value in this study and the measured value was 0.985 (p < 0.001) indicating that the method was reliable.
5. The results of the kernel density analysis showed that there were obviously three core areas of urban land use during the late Qing Dynasty: the North China Plain and the Central Plains urban core area; the Su-Hu, Zhejiang, and Anhui joint area; and the Chuan-Yu urban core area.

## Data Availability

All data generated or analysed during this study are included in this published article and its supplementary information files.
